# Pathogenic variants of *AIPL1*, *MERTK*, *GUCY2D*, and *FOXE3* in Pakistani families with clinically heterogeneous eye diseases

**DOI:** 10.1371/journal.pone.0239748

**Published:** 2020-09-25

**Authors:** Muhammad Rashid, Muhammad Qasim, Rafaqat Ishaq, Shazia Anwer Bukhari, Zureesha Sajid, Usman Ali Ashfaq, Asma Haque, Zubair M. Ahmed

**Affiliations:** 1 Department of Bioinformatics and Biotechnology, Government College University, Faisalabad, Pakistan; 2 Department of Otorhinolaryngology-Head and Neck Surgery, School of Medicine, University of Maryland, Baltimore, MD, United States of America; 3 University Institute of Biochemistry & Biotechnology, PMAS-Arid Agriculture University, Rawalpindi, Pakistan; 4 Department of Biochemistry, Government College University, Faisalabad, Pakistan; 5 Institute of Molecular Biology and Biotechnology, Bahauddin Zakariya University, Multan, Pakistan; University of Balochistan, PAKISTAN

## Abstract

Significant number out of 2.2 billion vision impairments in the world can be attributed to genetics. The current study is aimed to decipher the genetic basis of Leber congenital Amaurosis (LCA), Anterior Segment dysgenesis (ASD), and Retinitis Pigmentosa (RP), segregating in four large consanguineous Pakistani families. The exome sequencing followed by segregation analysis via Sanger sequencing revealed the LCA phenotypes segregating in families GCUF01 and GCUF04 can be attributed to c.465G>T (p.(Gln155His)) missense and novel c.139_140delinsA p.(Pro47Trhfster38) frameshift variant of *AIPL1* and *GUCY2D*, respectively. The c.1843A>T (p.(Lys615*) truncating allele of *MERTK* is homozygous in all the affected individuals, presumably suffering with RP, of the GCUF02 family. Meanwhile, co-segregation of the ASD phenotype and the c.289A>G (p.(Ile97Val)) variant of *FOXE3* was found in the GCUF06 family. All the identified variants were either absent or present in very low frequencies in the control databases. Our *in-silico* analyses and 3D molecular modeling support the deleterious impact of these variants on the encoded proteins. Variants identified in *MERTK*, *GUCY2D*, and *FOXE3* were categorized as “pathogenic” or “likely pathogenic”, while the missense variant found in *AIPL1* was deemed to have “uncertain significance” based upon the variant pathogenicity guidelines from the American College of Medical Genetics and Genomics (ACMG). This paper highlights the genetic diversity of vision disorders in the Pakistani population and reports the identification of four novel mutations in families who segregate clinically heterogeneous eye diseases. Our results give insight into the genotype-phenotype correlations of *AIPL1*, *FOXE3*, *MERTK*, and *GUCY2D* variants.

## Introduction

Hereditary vision impairment can result from a large group of complex eye disorders, including but not limited to retinal dystrophies (RD), optic neuropathy (e.g. glaucoma), lens diseases (e.g. cataract), and disorders related to eye development (e.g. anterior segment dysgenesis). These inherited diseases are generally featured by progressive and chronic visual impairment, genetic heterogeneity, and major clinical overlap among disorders. Many of these disorders also have high clinical and penetrance variability. Further complexity is attributed to their mode of inheritance, as these disorders can be inherited as autosomal recessive, autosomal dominant, X-linked, mitochondrial, and even through digenic and polygenic traits. Genetic studies in endogamous populations have been instrumental in characterizing the molecular basis of diverse vision disorders.

Pakistan has a rich anthropogeneological background and the highest prevalence of consanguineous marriages in the world [1]. The present study ascertained families from the Southern Punjab province in Pakistan with the aim to identify the underlying molecular basis of diverse segregating vision disorders. After clinical phenotyping, whole-exome sequencing was performed on four families, which revealed two novel and two reported variants of known vision impairment genes, *AIPL1*, *FOXE3*, *MERTK*, and *GUCY2D*. *AIPL1* encodes a molecular chaperon for the key phototransduction enzyme called Phosphodiesterase6 (PDE6), and its pathogenic variants are associated with Leber congenital amaurosis, dominant cone-rod dystrophy and recessive retinitis pigmentosa [2]. Transcription factor encoded by *FOXE3* has a forked head DNA binding domain, and variants of *FOXE3* are responsible for the dysgenesis of anterior segment tissues like lens and cornea resulting in microphthalmia, glaucoma, and aphakia [3]. While *MERTK* is one of the tyrosine kinase receptors of cell surface membrane expressed in retinal pigment epithelium. Recessive pathogenic variants of *MERTK* are reported in autosomal recessive retinitis pigmentosa [4]. Finally, GUCY2D encodes for the synthesis of enzyme photoreceptor guanylate cyclase GC-E, needed for the synthesis of second messenger of cGMP. Regulation of GUCY2D is by guanylate cyclase-activating proteins (GCAPs) through intra cellular Ca++ sensation. Pathogenic variants of *GUCY2D* are reported in association with LCA and dominantly inherited cone rod dystrophies [5]. The results of this study further support the utility of exome sequencing and genetic screening for families with diverse and clinically heterogenous segregating vision disorders in order to catalog the novel disease-causing variants of known genes. This will improve the clinical genetic diagnostic rate as well as help to establish the frequency of previously reported alleles in Pakistani population.

## Materials and methods

### Ascertainment and clinical phenotyping

The Institutional Review Board Committees at Government College University, Faisalabad, Pakistan and the University of Maryland, School of Medicine, MD, USA (IRB # HP-00061036) approved the current study. The study was done according to the principles of the Declaration of Helsinki and ARVO statement for research involving human subjects. Informed and written consent was obtained from all participating individuals. Clinical evaluations include comprehensive family history, physical examination, fundoscopy, slit lamp examination, visual acuity, and measurement of intraocular pressure via a tonometer. Genomic DNA was extracted from venous blood that was collected from all participants via our previously reported methods [6–8].

### Exome and sanger sequencing

Exome sequencing was performed on the probands of all families. Exome enriched genomic libraries were prepared using the Agilent SureSelect Human Expanded All Exon V6 kit and sequenced on an Illumina HiSeq4000 with an average coverage of 100X Variant calling, data alignment, and filtration were performed as described in our previous articles [6–8]. We used the Primer3 web resource (http://bioinfo.ut.ee/primer3-0.4.0/) to design primers for the selected variants for use in Sanger sequencing.

### Bioinformatic analyses and molecular modeling

Clustal Omega (https://www.ebi.ac.uk/Tools/msa/clustalo/) was used to align multiple sequences to appraise the evolutionary conservation of variants. While SIFT (https://sift.bii.a-star.edu.sg/), Polyphen-2 (http://genetics.bwh.harvard.edu/pph2/), Fathmm (http://fathmm.biocompute.org.uk/), and CADD scoring (https://cadd.gs.washington.edu/score) were used to evaluate the impact of the identified variants on the proteins which they encoded. Finally, the Varsome (https://varsome.com) online tool was used for the classification of variants as suggested by the American College of Medical Genetics and Genomics (ACMG).

To further evaluate the impact of variants on secondary structure, 3D protein models were generated through the web-based program I-TASSER (https://zhanglab.ccmb.med.umich.edu/I-TASSER/). Additionally, the online tool, UCSF CHIMERA (https://www.cgl.ucsf.edu/chimera/), was used to visualize the impact of amino acid change on protein folding and ionic interactions.

## Results

### Clinical phenotyping

**Fig 1:**
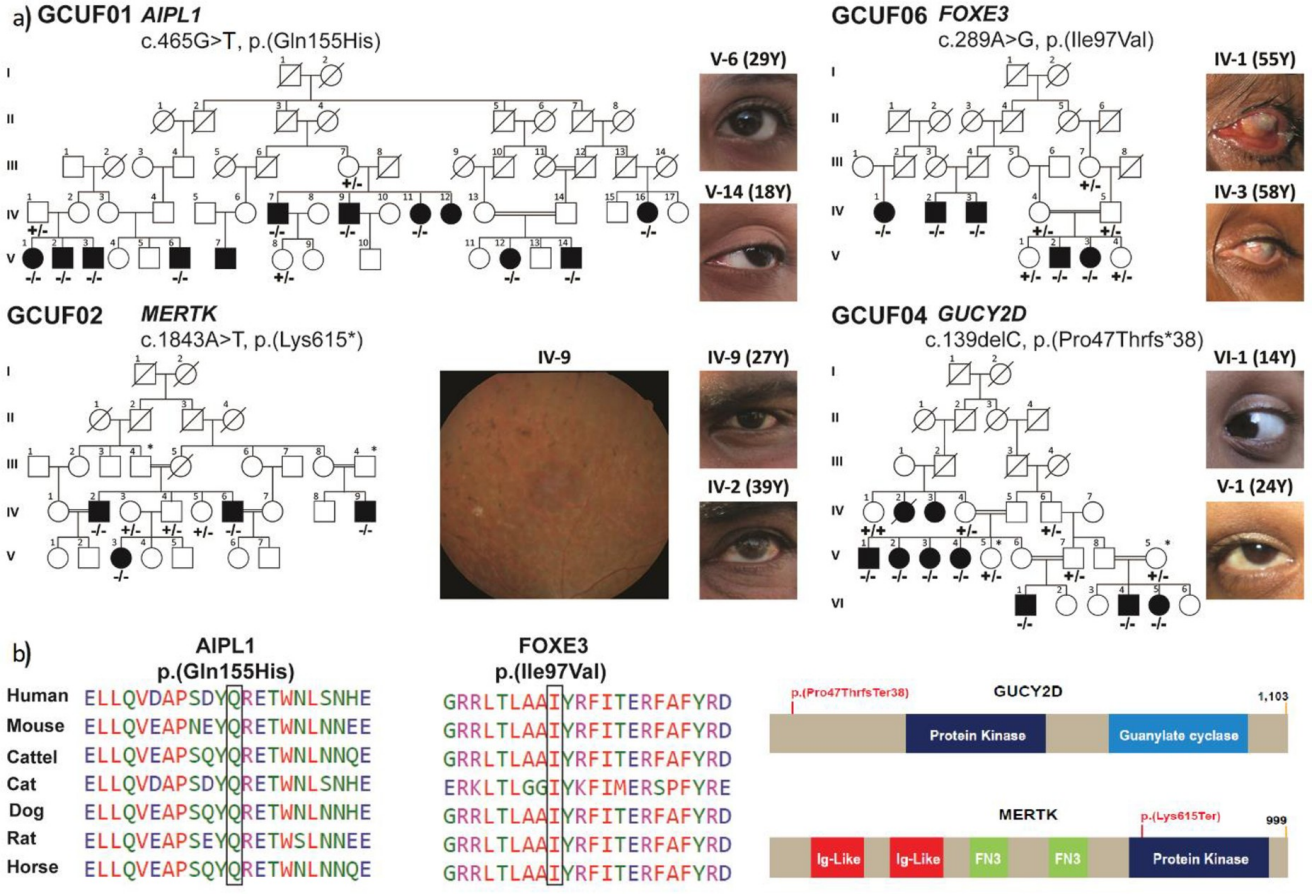
Family pedigrees of vision disorder and causative mutation. (**a**) Segregation of probable pathogenic alleles in four families from Pakistan having diverse visual disorders. Empty and filled symbols represent unaffected and affected individuals, respectively. Consanguineous marriages are indicated by double lines. The genotypes (homozygous, heterozygous, and wild type) of the identified alleles are also shown for each of the participating family members. Clinical phenotype/eye picture and fundus of the affected individual with (age) is shown against each pedigree. Asterisks (*) in pedigree drawings of families GCUF02 and GCUF04 mark the individuals drawn twice to show their relations with other family members. Images of the eyes of the affected individuals are also shown, along with the fundoscopic image of an affected individual of family GCUF02 to document the salt and pepper appearance of the retina due to retinitis pigmentosa. (**b**) Clustal-W sequence alignments of multiple amino acids of orthologous proteins shows evolutionarily conserved variant residues across different species for missense variants of AIPL1 and FOXE3. Protein truncation position of GUCY2D and MERTK is shown as stick diagram.

After obtaining IRB approval and informed consent from the four large consanguineous families, GCUF01, GCUF02, GCUF04, and GCUF06 (Fig 1A), we enrolled the families from Punjab, Pakistan. The neonatal clinical records of the affected participants were not available at the time of enrollment. Family history considered along with ophthalmological examinations reveal that the affected members of family GCUF01 have severe congenital nystagmus, an equal level of complete blindness irrespective of their ages (10–43 years) since their first year of life. Slit-lamp ophthalmoscopic and visual acuity studies reveal bony spicules in the retina. However, intraocular pressures (IOP) of family members were within the normal range (10–20 mm Hg, Table 1) at the time of testing. A tentative diagnosis of Leber congenital amaurosis (LCA) was assigned to the vision impairment phenotype segregating in family GCUF01. Similarly, phenotypic features were recorded in the affected individuals of family GCUF02, however with a key difference. In contrast to GCUF01, affected individuals of GCUF02 do not have congenital vision impairment. The youngest affected individuals (IV:9; 27 yrs) had sufficient perception of light during examination (Table 1). The pigmentary retinopathy of the retina (Fig 1A) was consistent with retinitis pigmentosa (RP).

**Table 1:** Family IDs, gender, age, clinical phenotypes, and ophthalmological remarks of participating individuals.

Family	ID	Gender	Age (Years)	IOP* mm of Hg OD/OS	Visual Acuity OD*/OS*	Remarks OD/OS
GCUF 1	IV:7	Male	40	12/16	NPL:NPL*	LCA*
	IV:9	Male	43	12/14	NPL:NPL	LCA
	IV:11	Female	32	12/12	NPL:NPL	LCA
	IV:16	Female	13	12/12	NPL:NPL	LCA
	V:1	Female	10	14/14	PL:PL*	LCA
	V:2	Male	13	14/12	NPL:NPL	LCA
	V:3	Male	19	14/18	NPL:NPL	LCA
	V:6	Male	29	14/12	NPL:NPL	LCA
	V:8	Female	12	14/18	6:10/6:6	Normal Vision
	V:12	Female	16	14/12	NPL:NPL	LCA
	V:14	Male	18	14/16	NPL:NPL	LCA
GCUF 2	IV:2	Male	39	14/12	NPL:NPL	Retinitis Pigmentosa
	IV:5	Female	36	14/18	6:6/6:10	Normal Vision
	IV:6	Male	55	14/16	NPL:NPL	Retinitis Pigmentosa
	IV:9	Male	27	14/12	PL:PL	Retinitis Pigmentosa
	IV:4	Female	13	14/16	PL:PL	Retinitis Pigmentosa
GCUF 4	IV:6	Male	70	12/18	6:6/6:10	Normal Vision
	V:1	Male	24	14/12	PL:PL	No Vision in Light; LCA
	V:2	Female	20	14/12	NPL:NPL	No Vision in Light; LCA
	V:3	Female	17	14/12	NPL:NPL	No Vision in Light; LCA
	V:4	Female	15	14/14	NPL:NPL	No Vision in Light; LCA
	VI:1	Male	14	14/16	NPL:NPL	No Vision in Light; LCA
	VI:4	Male	16	12/14	NPL:NPL	No Vision in Light; LCA
	VI:5	Female	12	14/16	NPL:NPL	No Vision in Light; LCA
GCUF 6	III:7	Female	65		6/24:6/28	Normal Vision
	IV:1	Female	55	NR	NPL:NPL	Corneal Haze, Microphthalmia
	IV:2	Male	55	NR	NPL:NPL	Corneal Haze Conus Cornea/Microphthalmia
	IV:3	Male	58	NR	NPL:NPL	Microphthalmia
	V:1	Female	15	12/12	6/6:6/6	Normal Vision
	V:2	Female	6	35/30	NPL:NPL	Conus Cornea, Corneal Haze
	V:3	Male	10	NR	NPL:NPL	Microphthalmia
	V:4	Male	25	12/14	6/6:6/6	Normal Vision

*IOP: Intra Ocular Pressure (Norma range: 10–12 mm of Hg); NPL: No Perception of Light; PL: Perception of Light NR: No Record; OD: Oculus Dextrus; OS: Oculus Sinister; LCA: Leber Congenital Amaurosis.

The affected members of GCUF04 have congenital nystagmus and photophobia with visual acuity of no finger count in daylight, except V:1 who was able to count fingers in daylight (Table 1). However, in low-light examination, they were able to recognize general objects. V:1 was even able to count fingers in daylight. Macular examination showed neovascularization and typical mottling of para-macular areas. An initial diagnosis of vernal keratoconjunctivitis in both eyes was made by the ophthalmologist who examined the family in Pakistan. In contrast to the above three families, the affected members of GCUF06 reported having congenital anterior segment dysgenesis (ASD), keratoconus, congenital bilateral corneal haze, and apparent microphthalmia (Fig 1A). The youngest affected female (V:2, 6 yrs) had an intraocular pressure of 30/35 (OD/OS) at the time of examination (Table 1).

### Genetic screening

Exome sequencing was performed for the proband of each family to determine the probable genetic cause(s) for the diversity among vision disorders segregating in the four families. Autosomal recessive inheritance (both homozygous and compound heterozygous) was assumed from the family trees. Four novel variants were prioritized through online prediction tools: c.465G>T, c.1843A>T, c.289A>G, and c.139delC in *AIPL1*, *MERTK*, *FOXE3*, and *GUCY2D*, respectively, (Fig 1A, Table 2). They were then validated using Sanger Sequencing in the available family members. These variants included two missense [p.(Gln155His), p.(Ile97Val)], one nonsense [p.(Lys615*)] and one frameshift allele p.(Pro47Thrfs*38)]. Variants identified in this study were present in evolutionarily conserved regions (Fig 1A) of the encoded proteins and were absent or had very low frequencies in gnomAD (Table 2).

**Table 2:** Genes, identified novel variants and their ACMG classification.

Family	Gene	Accession No.	cDNA change	Protein change	CADD	GnomAD	Polyphen2	SIFT	Fathmm	ACMG classification
GCUF1	AIPL1	NM_001033055	c.465G>T	p.(Gln155His)	20.9	0.00001627	Possibly damaging	Deleterious	Damaging	Uncertain Significance (PM2, PP3, BP1)
GCUF2	MERTK	NM_006343	c.1843A>T	p.(Lys615*)	41	0	N.A.	N.A.	N.A.	Pathogenic (PVS1, PM1, PM2, PP3)
GCUF4	GUCY2D	NM_000180	c.139_140delinsA	p.(Pro47Trhfster38)	N.A.	0	N.A.	N.A.	N.A.	Likely pathogenic (PVS1, PM2)
GCUF6	FOXE3	NM_012186	c.289A>G	p.(Ile97Val)	16.33	0.00002017	Deleterious	Deleterious	Damaging	Likely pathogenic (PM1, PM2, PM5, PP2, PP3)

**N.A.:** Not applicable

**CADD**: Combined Annotation Dependent Depletion, https://cadd.gs.washington.edu/

**gnomAD**: Genome Aggregation Database, http://gnomad.broadinstitute.org/

**PVS1**: Pathogenic very strong [null variant (nonsense, frameshift, canonical ±1 or 2 splice sites, initiation codon, single or multiexon deletion) in a gene here LOF is a known mechanism of disease)]

**PM1**: Pathogenic moderate 1 [Located in a mutational hot spot and/or critical and well-established functional domain (e.g., the active site of an enzyme) without benign variation]

**PM2**: Pathogenic moderate 2 [Absent from controls (or at extremely low frequency if recessive) in Exome Sequencing Project, 1000 Genomes Project, or Exome Aggregation Consortium]

**PM5**: Pathogenic Moderate 5 [Novel missense change at an amino acid residue where a different missense change determined to be pathogenic has been seen before]

**PP2**: Pathogenic supporting 2 [Missense variant in a gene that has a low rate of benign missense variation and in which missense variants are a common mechanism of disease]

**PP3**: Pathogenic supporting 3 [Multiple lines of computational evidence support a deleterious effect on the gene or gene product (conservation, evolutionary, splicing impact, etc.)]

**BP1:** Benign Supporting 1 [19 out of 23 non-VUS missense variants in the gene are benign]

**Fig 2:**
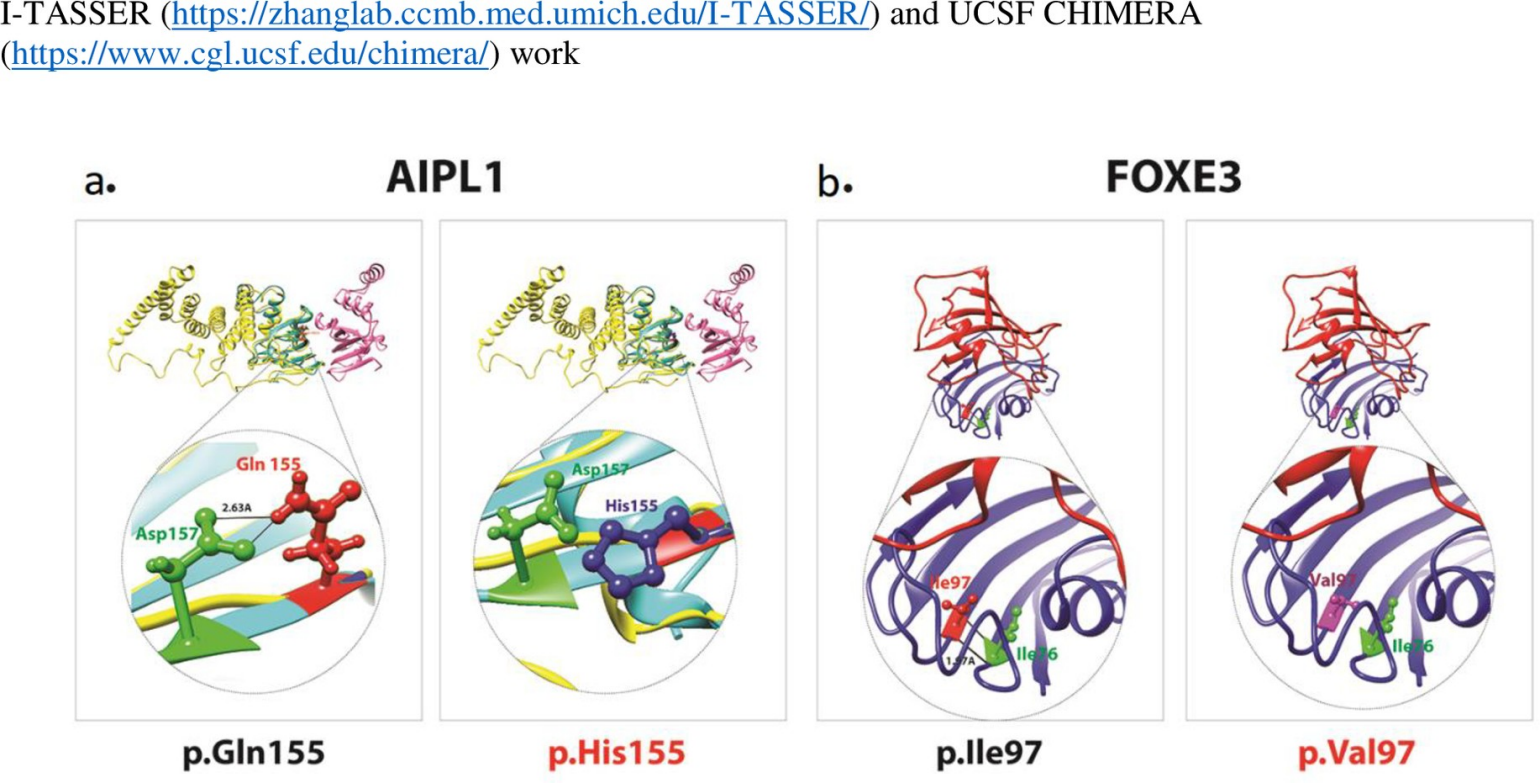
Protein 3D structural modeling of variants identified in this study. Structures of both wild type and mutated residues (red) are shown. Solid black lines represent the interactions with neighboring amino acids. Ball and stick representation in red shows wild type residue, while the blue and purple represent mutated amino acids. (a) The p.(Gln155His) variant of AIPL1 is predicted to cause loss of hydrogen bond with p.(Asp 157) impacting protein 3D folding and introduces torsion in the mutated protein. (b) The p.(Ile97Val) variant in FOXE3, is predicted to cause loss of ionic interaction and impact proper protein folding. The Isoleucine (Ile) at position 97 is predicted to form a hydrogen bond with p.Ile76, which is lost due to replacement with valine at position 97.

Next, to assess the predicted impact of identified variants on the encoded proteins secondary structures, we performed 3D molecular modeling with I-TASSER online programs. We concluded that the p.(Lys615*) nonsense variant of MERTK (Fig 2A) and the p.(Pro47TrhfsTer38) frameshift variant of GUCY2D are likely to yield complete loss of function of both proteins, as the mRNAs harboring these alleles will likely degrade through the nonsense-mediated decay (NMD) machinery [9]. In the unlikely event, MERTK mRNA escapes the NMD, the insertion of nonsense codon at amino acid position 615 is predicted to remove the carboxy tail. In contrast, the p.(Gln155His) variant, found in the GCUF01 family, is predicted to cause the loss of ionic interaction with aspartate residue at position 157 (Fig 2A), and therefore might impact the folding of the protein secondary structure of AIPL1. Neoconservative p.(Ile97Val) variant, located in the forkhead DNA binding domain of FOXE3 transcription factor, is also predicted to cause the loss of hydrogen bond with p.Ile97 (Fig 2B) and might impact DNA binding due to the change in its the domain folding.

## Discussion

High throughput sequencing (e.g. exome sequencing) has revolutionized genetics screening approaches for Mendelian disorders, including monogenic forms of vision disorders. Combinatorial approaches to identify individuals with actionable variants in highly penetrant monogenic forms of common vision disorders is absolutely essential if genomic medicine is to fulfill its promised purpose. Here, we report the identification of two novel and two reported disease-associated variants of *AIPL1*, *MERTK*, *FOXE3*, and *GUCY2D* in multiplex Pakistan families (Fig 1A). Identification of these variants is expected to aid in the accurate diagnosis of the vision disorder forms that segregate in the affected individuals, especially in the presence of limited clinical resources available in these families’ catchment areas in Pakistan.

The affected participants of the GCUF01 family are homozygous for c.465G>T (p.(Gln155His)) variant in *AIPL1* (Encoded protein: Aryl hydrocarbon receptor (AhR)-interacting protein-like 1 (AIPL1) with three tetratricopeptide motifs). AIPL1 acts as a molecular chaperon for the Phosphodiesterase6 (PDE6) key phototransduction enzyme [10]. Pathogenic variants in *AIPL1* are a common (5–10%) cause of LCA in diverse populations [11]. Severe congenital nystagmus and non-progressive vision loss together with a predicted pathogenic variant in *AIPL1* is consistent with the ophthalmologist diagnosis of LCA in the affected individuals of the GCUF01family.

Similarly, the RP phenotype in family GCUF02 is co-segregating with a truncating allele (p.(Lys615*)) of Mer tyrosine kinase (MERTK; OMIM#604705). MERTK belongs to a family (Tyro3/Axl/Mer) of tyrosine kinases [12] and is essential for the effective phagocytosis of photoreceptor outer segments by the retinal pigment epithelium [13–15]. Accumulation of cellular debris, due to impaired phagocytosis, hampers the supply of oxygen and nutrient to photoreceptor cells [16]. Pathogenic variants of MERTK are known to cause severe, faster, and early onset RP with worse macular degeneration [17]. The truncating (p.(Lys615*)) variant segregating in the GCUF02 family is predicted to truncate the carboxy tail of the encoded proteins, which would impact the kinase activity, which in turn is needed for the proper phagocytosis of photoreceptor outer segments engulfed by RPE [14, 16].

*GUCY2D* encodes a cyclic guanosine monophosphate (cGMP)-producing guanylate cyclase enzyme. Calcium and cGMP act as second messengers in phototransduction. The levels of cGMP in light-sensing photoreceptor cells are controlled by the cGMP-hydrolyzing phosphodiesterase and guanylate cyclase enzymes. Variants impacting the function of the GUCY2D enzyme lead to severe retinal dystrophies, including LCA type 1 and autosomal dominant cone-rod dystrophy [5]. The c.139delC truncating variant found in the GCUF04 family is expected to completely abolish the enzymatic activity of GUCY2D, which is consistent with the LCA phenotype observed.

Forkhead box protein E3 (FOXE3) belongs to family of forkhead transcription factors. A-helix of the forkhead motif of FOXE3 binds with a major groove of DNA during transcription [18]. Homozygous variants were found in inherited congenital primary aphakia, sclerocornea, with incomplete penetrance of glaucoma [19–21], while some dominant variants are associated with ocular dysgenesis, cataracts, and Peters’ anomaly [22, 23] in human. The “likely pathogenic” missense variant found in the GCUF06 family is associated with microphthalmia, sclerocornea, and anterior segment aplasia in most of the affected individuals, while one individual (V:2) also had high IOP (Table 1). Unavailability of FA, OCT, and ERG are the main limitations explaining genetic variants in relation to phenotypic diversity in this study. In summary, for families living in remote areas of Pakistan with limited economical resources and sparse health facilities, genetic screenings will likely help in complete diagnosis, family counseling, and disease management.
